# The Potential of Exercise on Lifestyle and Skin Function: Narrative Review

**DOI:** 10.2196/51962

**Published:** 2024-03-14

**Authors:** Ryosuke Oizumi, Yoshie Sugimoto, Hiromi Aibara

**Affiliations:** 1 Faculty of Nursing Shijonawate gakuen University Daito-shi Japan; 2 Osaka Metropolitan University Habikino-shi Japan; 3 Ehime University Toon-shi Japan

**Keywords:** skin function, lifestyle, exercise, reviews, knowledge synthesis, Review methods, review methodology, literature review, literature reviews, narrative review, narrative reviews, skin, dermatology, exercise, physical activity, fitness, lifestyles, smoking, diet, sleep, sugar intake, life habits, skin barrier

## Abstract

**Background:**

The skin is an important organ of the human body and has moisturizing and barrier functions. Factors such as sunlight and lifestyle significantly affect these skin functions, with sunlight being extremely damaging. The effects of lifestyle habits such as smoking, diet, and sleep have been studied extensively. It has been found that smoking increases the risk of wrinkles, while excessive fat and sugar intake leads to skin aging. Lack of sleep and stress are also dangerous for the skin’s barrier function. In recent years, the impact of exercise habits on skin function has been a focus of study. Regular exercise is associated with increased blood flow to the skin, elevated skin temperature, and improved skin moisture. Furthermore, it has been shown to improve skin structure and rejuvenate its appearance, possibly through promoting mitochondrial biosynthesis and affecting hormone secretion. Further research is needed to understand the effects of different amounts and content of exercise on the skin.

**Objective:**

This study aims to briefly summarize the relationship between lifestyle and skin function and the mechanisms that have been elucidated so far and introduce the expected effects of exercise on skin function.

**Methods:**

We conducted a review of the literature using PubMed and Google Scholar repositories for relevant literature published between 2000 and 2022 with the following keywords: exercise, skin, and life habits.

**Results:**

Exercise augments the total spectrum power density of cutaneous blood perfusion by a factor of approximately 8, and vasodilation demonstrates an enhancement of approximately 1.5-fold. Regular exercise can also mitigate age-related skin changes by promoting mitochondrial biosynthesis. However, not all exercise impacts are positive; for instance, swimming in chlorinated pools may harm the skin barrier function. Hence, the exercise environment should be considered for its potential effects on the skin.

**Conclusions:**

This review demonstrates that exercise can potentially enhance skin function retention.

## Introduction

The skin is the largest human organ that acts as a barrier between the body and the environment. Its role is to protect the body from the invasion of pathogens and to shield it from chemical and physical stimuli originating from the external environment; furthermore, it also prevents dehydration by mitigating water loss from the body [1,2]. The stratum corneum primarily serves as this barrier [1]. Skin functionality declines with age, a process evidenced not only in appearance, such as fine wrinkles, but also in quantitative indicators such as reduced skin elasticity and a decrease in the water content of stratum corneum [3-5].

A complex variety of factors, beyond age-related changes, influence the functional decline of the skin. Typical examples of influencing factors include lifestyle habits such as sun exposure, smoking, and diet [3,6,7]. Various studies have been conducted on the relationship between lifestyle habits and skin function. Specific lifestyle habits that are associated with skin function include daily moisturizing [8], bathing habits [8], stress [9,10], and sleep quality [11]. Improvement of these lifestyle habits is expected to prevent the functional decline of the skin.

In addition to the lifestyle habits mentioned above, exercise is gaining attention as a way to prevent skin dysfunction and improve aesthetics [12]. It is widely known that regular exercise not only aids in the prevention of various diseases but also plays a significant role in maintaining mental health [13-15]. However, the impact of exercise on skin function remains largely unexplored. In this review, we will briefly summarize the relationship between lifestyle and skin function and the mechanisms that have been elucidated so far. Additionally, we will introduce the expected effects of exercise on skin functionality.

## Methods

A review of the literature was conducted using PubMed and Google Scholar repositories for relevant literature published between 2000 and 2022 using the following keywords: exercise, skin, and life habits. The search was expanded to discover relevant literature on specific lifestyle habits. To discover relevant literature showing the effects of specific lifestyle habits on skin function, searches were conducted using the following keywords: smoking, dietary habits, ultraviolet light, hormones, and stress.

However, articles on the relationship between skin diseases such as atopic dermatitis and lifestyle habits were excluded.

## Results

### Skin Function

Preserving moisture and acting as a barrier are important functions of the skin [2]. These functions are mainly performed by the stratum corneum, which consists of keratinocytes stacked in a brick-like structure (brick and mortar model), with the cells being akin to bricks and intercellular lipids acting as the mortar, filling the spaces between the cells. These cells are further interconnected by desmosomes [2]. The natural moisturizing factors in the keratinocytes and intercellular lipids maintain skin hydration [16]. In addition, the dermis layer beneath the stratum corneum contains elastic fibers, such as collagen and elastin, which make the skin elastic and provide a barrier against physical stimuli [6]. When the skin’s moisturizing and barrier functions are compromised, it can lead to issues such as skin dryness and infections, which are caused by microorganisms entering the body’s defenses [17]. Hormones such as estrogen and growth hormones play an important role in maintaining these skin functions, including supporting skin elasticity and moisture retention. As we age, the secretion of these hormones declines, leading to a decrease in skin elasticity and hydration [18,19].

### Internal Factors Related to Skin Function

#### Aging

Age-related declines in skin functions, such as loss of elasticity, are explained by a decrease in collagen synthesis due to fewer fibroblasts in the dermis, a decrease in the number of sebaceous and sweat glands, and diminished blood flow to the skin [6,20,21]. These factors cause skin changes characteristic of older adults, such as coarse and dry skin, spots and dullness, wrinkles, and sagging. Various hypotheses have been made about the causes of age-related changes. Leading hypotheses include the generation of reactive oxygen species (ROS) or free radicals by normal endogenous metabolic processes, telomere shortening, and the accumulation of advanced glycation end products [22-25]. These age-related changes vary greatly in their intensity, depending on the individual’s race, personal characteristics, and different sites within the same person’s body [4,26]. The reasons for this variation are thought to be related to differences in the number of cells in the stratum corneum, the amount of melanin, and the amount of light exposure [26,27].

#### Hormone Balance (Estrogen and Growth Hormone)

Various hormones are associated with skin function. Of these, estrogen and growth hormones have been the focus of many studies as they are associated with age-related declines in skin functionality [17,28,29]. Changes in the secretion of these hormones occur with aging. The effects of decreased estrogen secretion on the skin are more pronounced in postmenopausal women because women secrete less estrogen after menopause [30]. Two important roles of estrogen for the skin are collagen production and wound healing [28,31]. Research has demonstrated that the decrease in estrogen levels associated with menopause results in reduced collagen levels in the skin. Conversely, estrogen replacement therapy in postmenopausal women has been shown to increase these collagen levels [32,33]. Collagen plays a crucial role in skin elasticity and skin thickness; consequently, a decrease in collagen content leads to skin wrinkling and thinning [28,31]. Although the direct relationship between skin elasticity and skin hydration is not clear, skin elasticity and skin hydration act as similar indicators of skin function, as skin elasticity and skin hydration decrease with reduced skin function [6]. The role of estrogen in wound healing is to suppress the inflammatory response and promote epithelialization in the wound [34]. The wound healing process encompasses several stages, starting with hemostasis and coagulation, followed by the inflammatory phase, and then the proliferative phase. An excessive inflammatory response can delay the inflammatory phase. Estrogen has been shown to regulate and suppress the inflammatory response in wounds [34,35]. In addition, since collagen production is active during the proliferative phase, estrogen administration has been shown to increase the amount of collagen in the wound and promote wound healing [34].

The secretion of growth hormone is involved in the synthesis of collagen [36]. It has been shown that excessive secretion of growth hormone causes thickening of the skin, while a deficiency in growth hormone causes skin thinning and a loss of elasticity [37,38]. In addition, growth hormone is involved in the development of sweat glands. An excess or deficiency in this hormone has been shown to cause excessive sweating or decreased sweating, respectively [19].

### External Factors Related to Skin Function

#### Sunlight

The most significant external factor affecting skin aging is ultraviolet radiation (UVR) from sunlight. Since most of the sun’s UVR (290-400 nm) is blocked by the Earth’s atmosphere, UVR reaching the Earth’s surface consists of >95% ultraviolet A (320-400 nm) and approximately 5% ultraviolet B (290-320 nm) [39,40]. UV energy is absorbed by skin cells and generates ROS that cause oxidative stress and damage various molecules, including DNA, in cells and tissues [39]. Additionally, UV light damages the collagen in the skin [41]. As a result, skin with prolonged and repeated exposure to sunlight becomes yellowish in tone, more stained, has an increase in fine and deep wrinkles, and loses its luster, becoming rough and dry [6].

#### Lifestyle Habits

##### Diet

Dietary habits refer to preferences for food and beverages, and various studies have revealed the effects of diet on the skin, albeit in rats.

One specific diet known to affect the skin is a high-fat diet. Dietary fat intake is closely related to the lipid composition of body adipose tissue and skin [42]. A high-fat diet can potentially induce oxidative stress and inflammatory responses in the skin, delay skin healing by decreasing protein synthesis, and cause morphological changes in the skin [43]. A close association has also been established between the consumption of sugars and fried foods and the acceleration of skin aging. The metabolism of sugars and proteins generates advanced glycation end products, and their accumulation accelerates skin aging [44]. Therefore, limiting the intake of sugars and proteins can be expected to delay skin aging [44].

Alcohol consumption may also expedite skin aging. Ethanol and acetone, byproducts of alcohol metabolism, may promote the proliferation of keratinocytes in the skin, thereby increasing its permeability and impairing its barrier function [45]. Additionally, the degree of facial aging increases in correlation with alcohol intake and time [46].

##### Smoking

Smoking is the most common lifestyle habit that adversely affects skin function. Studies examining the effect of past smoking history on current skin condition have shown that each pack-year increases the risk of wrinkle development by more than 5-fold. Additionally, smoking has also been shown to alter skin thickness and promote skin pigmentation [47-49]. Smoking constricts skin blood vessels and deteriorates skin blood flow, thereby reducing the oxygen supply to skin tissues. This promotes a decline in skin function, including a decrease in skin elasticity and skin hydration [50,51].

##### Stress and Sleep

The impact of stress and sleep quality on skin function has been evaluated using skin hydration and transepidermal water loss (TEWL) [9,11]. Stress can undermine the integrity of the stratum corneum by decreasing the production and secretion of lamellar body and keratinocyte proliferation, leading to a weakened skin barrier function and structural damage to the skin [52]. Stressed individuals have also been found to have delayed recovery from changes in TEWL due to stratum corneum removal by tape stripping [9]. The effects of sleep on the skin have been shown to occur in individuals with poor sleep quality and sleep deprivation. These individuals typically exhibit higher TEWL, reduced skin barrier function, and cosmetic changes [11,53,54]. These studies were cross-sectional or included results reported immediately after stressful exposure, and the effects of long-term stressful exposure on the skin are not clear.

### The Relationship Between Exercise and Skin

Exercise has been shown to increase cutaneous blood flow, with acute maximal exercise increasing the cutaneous blood perfusion total spectrum power density approximately 8-fold [55,56]. This is a physiological function of skin vasodilation, which is accompanied by an increase in skin temperature, to dissipate the heat generated by exercise [57]. The dilation of skin vessels is attenuated by nonexercise habits and aging, and it is affected by the moisture levels in the skin. Interestingly, there are no sex differences in the pattern of skin temperature changes [55,57-59]. However, regular exercise in older and postmenopausal women has been shown to improve cutaneous vasodilation by approximately 1.5-fold [60,61]. This is thought to be due to the increased responsiveness to nitric oxide in the dilation of cutaneous blood vessels [60]. In other words, having an exercise routine not only increases cutaneous blood flow during exercise but also improves cutaneous vasodilatory function. Since skin hydration occurs through a moisture gradient between the deeper layers and the surface of the skin, maintaining adequate skin blood flow is an important factor in preserving skin hydration [16]. Although there is no direct evidence that exercise promotes skin hydration, various cross-sectional studies have shown that the skin of regularly exercising adults and hospitalized older people is more hydrated than the skin of those who do not [62,63]. Additionally, exercise can reduce hot flashes in postmenopausal women [64]. Hot flashes are thought to be caused by a dysfunction in the body’s thermoregulatory control system [65], as well as vascular dysfunction [66]. Exercise has the potential to improve these functions.

Exercise can also improve age-related changes in sedentary older adults’ skin structure [67]. One possible cause of systemic dysfunction due to aging, including skin, is increased ROS production due to age-related mitochondrial dysfunction [68]. Exercise has received significant attention because it can prevent mitochondrial dysfunction and promote mitochondrial biosynthesis, thereby helping to prevent systemic functional decline [67,69]. It has been shown that exercise stimulates the secretion of interleukin-15, which activates mitochondrial biosynthesis in muscles. This mechanism is expected to prevent age-related changes in middle-aged women’s skin [69]. In fact, in mice that exercised, there was an improvement in skin structure due to an increase in the amount of collagen in the dermis layer. Moreover, it has been shown that when older adults exercise twice a week for 12 weeks, the stratum corneum of the skin, which has thickened with age, becomes thinner [67]. In middle-aged women, daily facial exercises for 8 weeks have caused cosmetic changes in facial appearance [70], and changes in skin structure can lead to cosmetic changes as well. Exercise also affects hormone secretion, including stimulating the secretion of growth hormone and estrogen [71-73]. As mentioned in the *Hormone Balance* section, growth hormone and estrogen are involved in the production of cutaneous collagen [28,36]. In the skin, collagen is involved in skin elasticity and skin thickness [28,31], and a decrease in collagen content leads to skin wrinkling and thinning. Therefore, it can be inferred that it may also affect skin elasticity and other factors. Future research is expected in these areas.

However, exercise does not always have a positive effect on the skin. An example of this is the risk of skin eczema due to the composition of swimming pools [74,75]. Although this is not a direct effect of swimming exercise, it has been suggested that the chlorine used in pool disinfection may damage the skin barrier function [76]. Therefore, it is necessary to consider the possibility that the environment in which exercise is performed may adversely affect the skin.

Finally, Table 1 shows which exercises improve skin function.

**Table 1:** Exercise needed to improve skin function.

Improved skin function	Exercise details
Skin blood flow	Acute maximal exercise [55,56] Aerobic Training [56]
Vasodilator function in cutaneous microvessels	Aerobic Training [60,61]
Moisturizing function	Daily activity level [62,63]
Postmenopausal hot flushes	Moderate-intensity exercise training [64]
Structural of skin	Endurance exercise [67]
Facial appearance	Facial exercise [69]

## Discussion

Skin aging is a complex and lengthy biological process influenced by genetic and environmental factors. Although there are various therapeutic approaches to combat skin aging, such as hyaluronic acid injections and hormone replacement therapy, each method has its drawbacks. With people’s increasing demands for effective, safe, and sustainable treatment methods, the prevention and mitigation of skin aging through lifestyle management has become a trend.

It is undeniable that the skin is affected by lifestyle habits, and a consensus exists around lifestyle habits that negatively affect skin function. However, numerous questions remain unanswered regarding the effects of dietary and stress-coping interventions on skin function. This is likely due to ethical issues and the lack of guaranteed uniformity in clinical experimental conditions, which can lead to ambiguous results.

Exercise interventions have a relatively small potential for ethical problems and can have uniform experimental conditions. This review demonstrates that exercise can potentially enhance skin function retention. Although the design of the studies that were conducted varied, it was clear that exercise increases skin blood flow, increases keratin water content, and changes skin structure. The effects of exercise on the skin have previously been shown piecemeal, but this review has allowed us to synthesize the findings. These findings suggest the effectiveness of habitual exercise in improving skin function. Future studies should investigate the effects of exercise on the skin under different experimental conditions, such as varied exercise content and duration, as well as the physiological mechanisms involved.

## Abbreviations

ROS: reactive oxygen species
TEWL: transepidermal water loss
UVR: ultraviolet radiation
